# Supplementary material for: Process bigraphs and the architecture of compositional systems biology
Source: PLoS Comput Biol. 2026 Oct 9;22(10):e1014819. doi: 10.1371/journal.pcbi.1014819 (PMC13652326; doi:10.1371/journal.pcbi.1014819)
Supplement: Supplementary file 1 [file pcbi.1014819.s001.pdf]

Supplement #1 for

Process Bigraphs and the Architecture of Compositional Systems Biology

Eran Agmon *

Ryan K. Spangler

Center for Cell Analysis and Modeling,
University of Connecticut Health,
Farmington, Connecticut, USA

August 13, 2026

Contents

1 Introduction **2**

2 Vivarium 2.0 software suite **2**

3 Process bigraph semantics **3**

3.1 Mathematical preliminaries 3
3.2 Serialized representation 3
3.3 Types, schema trees, and state trees 4
3.3.1 Type engine and type registry 5
3.4 Links, ports and wiring 6
3.5 Processes and delta semantics 6
3.5.1 Process nodes and the link registry 6
3.5.2 Deltas and type-directed application 7
3.5.3 Single-process update protocol 9
3.5.4 Worked example: single-process update 9
3.6 Explicit definition of a process bigraph 10
3.7 Composites 11
3.8 Orchestration 12
3.8.1 Event times and schedule 12
3.8.2 Small-step orchestration semantics 12
3.8.3 Update reconciliation 13
3.8.4 DAG-structured discrete workflows 14

*Correspondence: agmon@uchc.edu

3.8.5 Recursive composition . . . 14
3.8.6 Large-scale orchestration . . . 14
3.9 Emitters and result capture . . . 15
3.10 Protocols . . . 15

1 Introduction

This supplementary materials document for "Process Bigraphs and the Architecture of Compositional Systems Biology" provides the technical underpinnings of the Process Bigraph framework introduced in the main text. Our goals are to (i) make the semantics of process bigraphs precise, (ii) connect these abstract definitions to concrete Python classes in the Vivarium 2.0 software stack, and (iii) illustrate the framework on a fully worked example (Spatio-Flux).

We proceed with the following sections:

- Section 2 introduces the Vivarium 2.0 software suite, the core libraries used in this work, and the main runtime classes we refer to in the rest of the supplement.
- Section 3 develops the formal, schema-based semantics of process bigraphs in an implementation-agnostic way, but with direct correspondence to the classes and type registries provided by the software libraries.
- Supplementary Materials #2 gives a detailed Spatio-Flux instantiation of these semantics, showing how field dynamics, stochastic/deterministic particle movement dynamics, and coupled particle–field metabolism are represented as process bigraphs and executed by the Vivarium 2.0 stack.

2 Vivarium 2.0 software suite

The Vivarium 2.0 software suite provides the reference implementation of the Process Bigraph framework used in this paper. It consists of three core libraries and an application repository:

- `bigraph-schema`: a foundational library that defines a compositional type system and hierarchical data structures using JSON-based schemas. It provides the *type engine* (e.g. `Core`) and a global `type_registry` that together implement: (i) schema compilation to path-addressed schema trees, (ii) validation of state trees, and (iii) type-specific update operators `apply` used in the delta semantics of Section 3.
- `process-bigraph`: the dynamic core of the reference implementation, and the library identified as "the `process-bigraph` library" throughout this supplement. It defines:
  - the `Process` and `Step` classes, which implement typed *process nodes* with input/output ports, configuration, and an `address` used to look up their implementation;
  - the `Composite` class, which encapsulates an entire process bigraph as a single process with an explicit interface;
  - the event-scheduling and orchestration logic that maintains a global time, an update schedule, and the repeated evaluation of process handlers.
- `bigraph-viz`: a visualization library that parses the JSON representation of a process bigraph and renders it as a graph.

In addition to the core libraries, we use domain-specific application and tooling repositories:

- `spatio-flux`: a complete multiscale example built on `process-bigraph` and `bigraph-schema` that defines `Process` and `Composite` subclasses for metabolic processes, field dynamics, particle dynamics, and coupled particle–field interactions. The repository includes an extended test suite

that runs dozens of process bigraph documents, some of which are referenced in Supplement 2 as concrete instances of the abstract semantics.

- `viva-superpowers`: a repository of reusable AI agent skills for scaffolding process wrappers, adapters, and composite connection patterns. It is used to accelerate the integration of legacy simulators into the Process Bigraph framework.

All source code, documentation, tutorials, and examples are available on the Vivarium Collective GitHub:

- `process-bigraph`: `https://github.com/vivarium-collective/process-bigraph`
- `bigraph-schema`: `https://github.com/vivarium-collective/bigraph-schema`
- `bigraph-viz`: `https://github.com/vivarium-collective/bigraph-viz`
- `spatio-flux`: `https://github.com/vivarium-collective/spatio-flux`
- `viva-superpowers`: `https://github.com/vivarium-collective/viva-superpowers`

3 Process bigraph semantics

This section develops the formal, schema-based semantics of process bigraphs, expressed in a set-theoretic, function-based language. We begin with mathematical preliminaries that define paths, schema trees, state trees, and typed update operations. These abstractions are then grounded in the running Michaelis–Menten example introduced in Fig. S1, with a worked single-process update given in Section 3.5.4. From there, we proceed to links, ports, wiring, processes, composites, and orchestration. Supplement 2 then mirrors this general account with a Spatio-Flux-specific instantiation, so that readers can move back and forth between abstract definitions and concrete examples.

3.1 Mathematical preliminaries

A bigraph is organized as a hierarchical *tree* (or, more generally, a forest) whose nodes are addressed by finite paths. This representation matches naturally with JSON: objects correspond to nodes with children keyed by field names, arrays correspond to nodes with children keyed by integer indices, and following a sequence of keys or indices selects a unique location in the structure.

Let $\mathcal{M}$ be the set of *marks* (atomic path components), and let

$$\mathcal{P} = \mathcal{M}^*$$

be the set of finite paths, implemented as lists of marks (e.g. `["organism", "cell", "mass"]`).

Any JSON-like hierarchical structure induces a tree of such paths: objects and arrays correspond to internal nodes, and primitive values correspond to leaves. In this supplement, both schemas and states are treated as partial maps from paths in $\mathcal{P}$ to types or values, respectively.

We can fix:

- a set of types $\mathcal{T}$,
- a set of values $\mathcal{V}$,
- a subset of paths $\mathsf{StorePath} \subseteq \mathcal{P}$ that designate locations where concrete values may be stored.

3.2 Serialized representation

A process bigraph may appear in three equivalent textual forms (Fig. S1):

1. a JSON-like serialized document, which is the canonical stored format;
2. a reduced tree notation, used in this supplement for readability;

3. a linearized one-line syntax using bracketed annotations.

These formats differ only in presentation. JSON and the linearized syntax can be parsed by the `Core` engine into the same in-memory executable composite, and round-trip conversion between them is deterministic and lossless. The reduced tree notation is a human-readable rendering of the same structure, used here for clarity.

```
{
  "schema": {
    "stores": {
      "substrate_conc": "float[mM]",
      "enzyme_conc": "float[mM]",
      "product_conc": "float[mM]"
    },
    "processes": {
      "mm_process": {
        "inputs": {
          "substrate": "float[mM]",
          "enzyme": "float[mM]"
        },
        "outputs": {
          "product": "float[mM]"
        }
      }
    }
  },
  "state": {
    "stores": {
      "substrate_conc": 1.0,
      "enzyme_conc": 0.5,
      "product_conc": 0.0
    },
    "processes": {
      "mm_process": {
        "inputs": {
          "substrate": ["stores",
"substrate_conc"],
          "enzyme": ["stores", "enzyme_conc"]
        },
        "outputs": {
          "product": ["stores", "product_conc"]
        }
      }
    }
  }
}
```

```
schema:
  stores:
    substrate_conc: float[mM]
    enzyme_conc: float[mM]
    product_conc: float[mM]
  processes:
    mm_process:
      inputs:
        substrate: float[mM]
        enzyme: float[mM]
      outputs:
        product: float[mM]
state:
  stores:
    substrate_conc: 1.0
    enzyme_conc: 0.5
    product_conc: 0.0
  processes:
    mm_process:
      inputs:
        substrate: [stores, substrate_conc]
        enzyme: [stores, enzyme_conc]
      outputs:
        product: [stores, product_conc]
```

Figure S1: **Equivalent representations of a simple Michaelis–Menten process bigraph.** *Left:* JSON-like serialized document. *Right:* Reduced tree notation used throughout this supplement. The schema declares three concentration stores using unit-parameterized `bigraph-schema` types, such as `float[mM]`, and a process interface with two inputs (`substrate`, `enzyme`) and one output (`product`). The state assigns concrete store values and wires the process ports to their corresponding store paths. This same example is reused below to illustrate typed process interfaces, wiring, delta generation, and type-directed update application.

3.3 Types, schema trees, and state trees

A *schema tree* is a partial map

$$\Sigma : \mathcal{P} \rightharpoonup \mathcal{T},$$

assigning a type to each path in a JSON-like hierarchical structure. Each $\Sigma(p)$ specifies the type governing the subtree rooted at p. Composite types appear at internal nodes and constrain the structure of their children. Leaf types specify primitive values such as numbers, strings, or booleans. A schema tree therefore determines the allowed structure of a process bigraph: its stores, its process interfaces, its configuration fields, and the types of all values that may appear in the state.

A *state tree* is a partial map

$$x : \mathcal{P} \rightharpoonup \mathcal{V},$$

assigning concrete values to paths. Leaves at designated store paths correspond to the *stores* of the system. Internal nodes organize these stores, process nodes, configuration values, and wiring information into a single hierarchical state.

Figure S1 provides the running example used throughout this section. In the schema portion of the figure, the stores `substrate_conc`, `enzyme_conc`, and `product_conc` are all declared with the unit-parameterized type `float[mM]`. This notation indicates that the base value type is `float`, while the bracketed parameter records the unit associated with the value. The same schema also declares a Michaelis–Menten process interface, with typed input ports `substrate` and `enzyme`, and a typed output port `product`.

The state portion of Fig. S1 then assigns concrete values to the stores: `substrate_conc` is initialized to `1.0`, `enzyme_conc` to `0.5`, and `product_conc` to `0.0`. The state also records how the process ports are wired to those stores. For example, the `substrate` input port is wired to `[stores, substrate_conc]`, and the `product` output port is wired to `[stores, product_conc]`.

A state tree x is *well-formed* for a schema tree Σ when, for every path p where both are defined, the value $x(p)$ is valid for the type declared at $\Sigma(p)$. We write this as the typing judgment

$$\Sigma \vdash x : \mathsf{State},$$

which is read as: under the schema Σ, the state tree x is a well-formed state. In Fig. S1, this means that the numeric values stored at concentration paths are valid values for the declared type `float[mM]`, and that the process ports are connected to stores with matching types.

3.3.1 Type engine and type registry

The type engine, implemented by `bigraph-schema` through the `Core` class, maintains a global `type_registry`, written R_T. This registry defines the available types and their semantics. For each type, the registry may specify:

- how values of that type are parsed and validated,
- how unit-parameterized type expressions such as `float[mM]` are interpreted,
- how updates are applied through a type-specific operator apply_τ,
- how values are serialized and deserialized.

Given a schema tree Σ and state tree x, the type engine checks that the typing judgment

$$\Sigma \vdash x : \mathsf{State}$$

holds. In the Michaelis–Menten example, this includes checking that each concentration store contains a valid floating-point value and that each process port is wired to a store with a compatible type.

3.4 Links, ports and wiring

In `bigraph-schema`, a *link* is a node whose schema specifies a typed interface. A process node is a specialized link: it has input ports, output ports, and runtime information that determines how the process is executed.

Figure S1 shows a minimal example. The process node `mm_process` declares two input ports, `substrate` and `enzyme`, and one output port, `product`. Each port is typed as `float[mM]`, matching the concentration stores to which the ports are connected.

Formally, for a link at path $p \in \mathcal{P}$, the schema tree Σ determines:

- its interface, with input ports $P_{\text{in}}(p)$ and output ports $P_{\text{out}}(p)$,
- for each port ℓ, the declared type $\tau_\ell = \Sigma(p.\ell)$.

The corresponding state tree records the wiring of each port. A link node in the state tree x stores:

- `inputs`: a mapping from input-port names to the state paths where their values are read,
- `outputs`: a mapping from output-port names to the state paths where their updates are written,
- `config`: process-specific parameters, when needed by the process implementation.

For the Michaelis–Menten example in Fig. S1, the input port `substrate` is wired to `[stores, substrate_conc]`, the input port `enzyme` is wired to `[stores, enzyme_conc]`, and the output port `product` is wired to `[stores, product_conc]`.

From these `inputs` and `outputs` fields, the global wiring pattern is obtained. The wiring map is therefore not stored as a separate object. It is derived from the collection of link nodes in the state tree.

Formally, collecting these entries across all link paths yields:

$$W : (\mathsf{LinkPath} \times \mathsf{PortName}) \to \mathcal{P},$$

where $W(p, \ell)$ returns the state-tree path connected to port ℓ of the link at p, and $\mathsf{LinkPath}$ is the set of paths whose schema entries denote links or their specializations, such as `process`, `step`, or `composite`.

Wiring is *well-typed* when each port is connected to a state path whose schema type matches the port's declared type. For instance, the `substrate` input of `mm_process` is well-typed because it is declared as `float[mM]` and is wired to the store `substrate_conc`, which is also typed as `float[mM]`.

3.5 Processes and delta semantics

3.5.1 Process nodes and the link registry

`process-bigraph` extends the generic typed-link mechanism of `bigraph-schema` by introducing `Process`, `Step`, and `Composite` as specialized link types. Like all links, a process node has typed input and output ports and participates in the global wiring map W. In addition, a process node carries the information needed for runtime execution.

The most important runtime fields are:

- an `address`, identifying the concrete implementation of the process,
- a `config` subtree, containing process-specific parameters,
- an `interval`, declaring the process's nominal time advance,
- typed input and output ports, inherited from the schema.

The *link registry*

$$R_L : \mathsf{Address} \to \mathsf{HandlerClass}$$

maps each address to a concrete handler class. During initialization, the runtime uses the process address and configuration to instantiate the handler. The schema determines what kind of process may appear; the handler instance determines how that process behaves when it is executed.

Process interfaces as typed function signatures. At the schema level, every process node specifies a typed interface: the values it can read and the values it can update. This can be written as a function signature:

$$\mathsf{process}_{p_{\mathrm{proc}}}\big(\mathrm{config}^{\tau_c}\big) : \big(\mathrm{in}_1^{\tau_{i1}}, \mathrm{in}_2^{\tau_{i2}}, \dots\big) \;\longrightarrow\; \big(\mathrm{out}_1^{\tau_{o1}}, \mathrm{out}_2^{\tau_{o2}}, \dots\big).$$

In Fig. S1, the Michaelis–Menten process has two inputs of type `float[mM]` and one output of type `float[mM]`. Thus, its process contract says that it may read substrate and enzyme concentrations and produce an update to product concentration.

Processes as delta generators. At runtime, the instantiated handler implements this contract through an `update` method. Abstractly, this has the form:

$$\mathsf{update} : \big(\mathrm{config}^{\tau_c}, \mathrm{in}_1^{\tau_{i1}}, \dots\big) \;\longrightarrow\; \Delta.$$

The result Δ is a *delta*: a description of how the state should change. For a process with several output ports, the delta is usually a tree-structured object whose top-level branches correspond to those output ports. For the Michaelis–Menten example, the relevant output branch is `product`; the value under that branch is the increment that should be applied to `product_conc`.

3.5.2 Deltas and type-directed application

For each type $\tau \in \mathcal{T}$, the type engine defines a type-specific update operator

$$\mathsf{apply}_\tau : \mathcal{V}_\tau \times \Delta_\tau \to \mathcal{V}_\tau,$$

where Δ_τ is the class of deltas valid for τ. Deltas describe *how* a value should change. The type determines what form the delta may take and how it is applied.

For example, the type `float[mM]` uses the update semantics of the underlying `float` type. A scalar delta is interpreted additively:

$$x' = x + \delta.$$

Thus, if `product_conc` currently has value `0.0` and the Michaelis–Menten process produces the delta `0.08`, then the updated value is `0.08`.

Representative examples of type-specific update operators are shown in Table S1.

Apply function	Delta type	Semantics
`apply_float`	$\Delta \in \mathbb{R}$	Additive update of a scalar value: $x \leftarrow x + \Delta$. Unit-parameterized forms such as `float[mM]` use the same scalar update semantics.
`apply_array`	$\Delta \in \mathbb{R}^{n \times m}$ or sparse index updates	Element-wise additive update of an array: $X \leftarrow X + \Delta$. Sparse deltas may target specific indices without requiring a full dense array update.
`apply_conc_counts`	$\Delta n \in \mathbb{R}$	$\text{apply}_{\text{conc\_counts}}((c, n), \Delta n) = \left(\frac{n+\Delta n}{V}, n + \Delta n\right)$, where n is the molecular count and $c = n/V$ is maintained as an invariant.
`apply_map`	$\Delta : K \rightharpoonup \Delta_\tau$ with optional `_add`/`_remove`	Key-wise update of a map: for each k, $x_k \leftarrow \mathsf{apply}_\tau(x_k, \Delta(k))$. Structural map updates may additionally insert or remove keys through `_add` and `_remove` operations.

Table S1: Examples of type-specific update operators apply_τ used by the type engine.

We distinguish three broad categories of deltas.

1. Primitive deltas. Primitive deltas are leaf-level updates applied to non-structured values. For example, a scalar concentration store may receive a numeric increment. If the current value is x and the delta is δ, the updated value is:

$$x' = \mathsf{apply}_\tau(x, \delta).$$

2. Composite deltas. Composite deltas are tree-structured updates. They are used when a process updates a structured value, such as a record, array, map, or a process with multiple output ports. A composite delta may contain primitive deltas at its leaves. For example, a process with outputs `product` and `heat` could return a delta with one branch for each output port. The wiring map then routes each branch to the appropriate store.

3. Structural deltas. Structural deltas modify the shape of the state tree or schema tree. They include operations such as:

- **insert**: add a new subtree,
- **delete**: remove a subtree,
- **move**: relocate a subtree,
- **rewrite**: modify fields, types, shapes, or metadata,
- **rewire**: modify `inputs` or `outputs` so ports point to different stores.

Because processes, ports, configuration fields, schedule entries, and wiring information are all represented as subtrees, structural deltas can express graph-level changes such as division, merging, engulfment, spawning, or rewiring. These graph changes are not handled by a separate mechanism. They are expressed as typed deltas and applied through the same type-directed update semantics as ordinary numerical updates.

A general form of structural delta is provided by the `BigraphicalReactiveSystem` process included in the `process-bigraph` library. When wired to a point in the place graph, this process applies bigraphical reactions in the sense of Milner [**milner2009space**]. Each reaction is specified

by a pair of bigraphs: a *redex*, which defines the structural pattern to be matched, and a *reactum*, which defines the structure that replaces the matched region.

Operationally, the reactive-system process searches the subtree to which it is wired for occurrences of the redex. When a match is found, the matched region is rewritten according to the reactum, yielding a structural delta on the process bigraph. This extends the basic container-level structural operations, such as `_add` and `_remove`, to the full range of transformations expressible by classical bigraphical reactive systems. For example, reactions can move nodes to different levels of the place graph, add or remove links or nodes under specified conditions, or replace one structured configuration with another.

Reactive-system processes therefore make graph rewriting available as an ordinary process within the same execution framework. Users may combine numerical processes, workflow steps, simulation wrappers, and bigraphical reactions in a single process bigraph. In this view, primitive and composite deltas update values within existing stores, while reactive-system structural deltas update the organization of stores, processes, ports, links, and nested structure.

3.5.3 Single-process update protocol

A single update step for a process located at path p_{proc} has three stages.

1. Input projection. The runtime first uses the wiring map W to read the values connected to the process input ports. For each input port ℓ,

$$x_{\text{in}}(\ell) = x\big(W(p_{\text{proc}}, \ell)\big).$$

2. Evaluation. The process handler is then called with the projected input values and the timestep Δt chosen by the orchestration policy:

$$\delta = \mathsf{update}_{p_{\text{proc}}}(x_{\text{in}}, \Delta t).$$

The result is a delta whose structure follows the process output ports.

3. Application. Finally, each output delta is routed through the wiring map to its target store. For each output port ℓ, let

$$q = W(p_{\text{proc}}, \ell)$$

be the target state path, and let $\tau = \Sigma(q)$ be the type declared for that path. The type engine applies the delta using:

$$x'(q) = \mathsf{apply}_{\tau}\big(x(q),\, \delta(\ell)\big).$$

Applying all output deltas yields a new state tree x' that remains well-typed with respect to the schema Σ.

3.5.4 Worked example: single-process update

We now walk through one update of the Michaelis–Menten process from Fig. S1. The initial state contains three stores:

$$\texttt{substrate\_conc} = 1.0, \qquad \texttt{enzyme\_conc} = 0.5, \qquad \texttt{product\_conc} = 0.0,$$

all typed as `float[mM]`. The process `mm_process` has two input ports, `substrate` and `enzyme`, and one output port, `product`.

During input projection, the runtime follows the wiring stored in the state tree and reads

$$\texttt{substrate} = x(\texttt{[stores, substrate\_conc]}) = 1.0, \qquad \texttt{enzyme} = x(\texttt{[stores, enzyme\_conc]}) = 0.5.$$

These values are passed to the Michaelis–Menten handler, which computes an update for the product output. For example, suppose that over the current timestep it returns

$$\delta(\texttt{product}) = 0.08.$$

This delta says that the product concentration should increase by `0.08`.

The runtime then follows the output wiring,

$$W(\texttt{mm\_process}, \texttt{product}) = \texttt{[stores, product\_conc]},$$

and applies the delta to the target store. Because `product_conc` has type `float[mM]`, the type engine uses scalar additive update semantics:

$$\texttt{product\_conc}' = \mathsf{apply}_{\texttt{float[mM]}}(0.0, 0.08) = 0.08.$$

After the update, the state contains

$$\texttt{substrate\_conc} = 1.0, \qquad \texttt{enzyme\_conc} = 0.5, \qquad \texttt{product\_conc} = 0.08.$$

Thus, one process update consists of reading wired inputs, calling the process handler, routing the output delta to the wired store, and applying the update according to the store's declared type.

3.6 Explicit definition of a process bigraph

We can now summarize the core ingredients of the framework as a single structure.

A *process bigraph* is a tuple

$$B = (\Sigma, x, R_T, R_L),$$

consisting of:

1. $\Sigma : \mathcal{P} \rightharpoonup \mathcal{T}$, a schema tree assigning a type to each path in a JSON-like hierarchy;
2. $x : \mathcal{P} \rightharpoonup \mathcal{V}$, a state tree assigning concrete values to paths, including the `inputs`, `outputs`, and handler instances stored at link and process nodes;
3. R_T, the type registry providing validation and type-directed update operators apply_τ for each type τ;
4. R_L, the link registry mapping process addresses to handler classes, from which configured process instances are created and stored in x.

A global wiring map W is *derived* from the `inputs` and `outputs` fields stored in the state tree x for each link node.

These components must satisfy the following consistency conditions:

- **Type correctness:** The typing judgment $\Sigma \vdash x : \mathsf{State}$ holds: whenever both $\Sigma(p)$ and $x(p)$ are defined, $x(p)$ is valid for the type declared at $\Sigma(p)$.
- **Well-typed wiring:** For every link node and port ℓ, the schema type declared for that port matches the schema type at its destination path obtained from the derived wiring map W.
- **Well-formed processes:** Each process node in $\mathsf{LinkPath}$ has a valid `address` pointing to a handler class in R_L, and the state tree contains a corresponding configured handler instance stored at the same path.

A process bigraph is therefore a fully typed, wired, and executable structure, ready to be advanced in time by the orchestration semantics described in Section 3.8.

3.7 Composites

A `Composite` is a specialized `process` that serves as the main simulation engine of the `process-bigraph` library. Its configuration encodes an entire process bigraph — a schema tree, a state tree, and the registries needed to interpret them — so that a composite behaves as a full, self-contained simulation when executed. An example is shown in Fig. S2.

A composite is also a process, that connect its internal process bigraph to an external state. It does this with two addition attributes:

- declares an `interface`, consisting of typed input and output ports just like any other process node;
- declares a `bridge` mapping these external ports to internal state-tree paths within the embedded bigraph.

The interface specifies the composite's external API, while the `bridge` acts as the inward-facing wiring. The type of each interface port must match the schema type at its internal bridge destination.

At runtime, the composite's configuration stores:

- the embedded schema tree Σ_{state},
- the embedded state tree x_{state}, including all internal link and process nodes,
- the global `type_registry` R_T,
- the global `link_registry` R_L, providing handler classes for processes.

As in the top-level case, the internal wiring pattern is not stored separately: it is *derived* from the `inputs` and `outputs` fields of link nodes within x_{state}.

Operationally, a composite C is identified as

$$C = \big(\Sigma_{\text{state}},\ x_{\text{state}},\ R_T,\ R_L,\ \textsf{bridge},\ \textsf{interface}\big),$$

with internal wiring implicitly determined by x_{state}.

Under orchestration (Section 3.8), a composite behaves as a standard process externally: values arriving at its interface ports are routed inward through the `bridge`, internal processes advance according to their scheduling rules, and outgoing deltas are returned through the external ports. Composites thus provide hierarchical modularity: externally a single process, internally a complete process bigraph.

```
process1:
  outputs:
    port1:  [store1.1]
    port2:  [store1.2]

process2:
  outputs:
    port1:  [store1.1]
    port2:  [store1.2]

bridge:
  inputs:
    port1:  [store1.1]
    port2:  [store1.2]
```

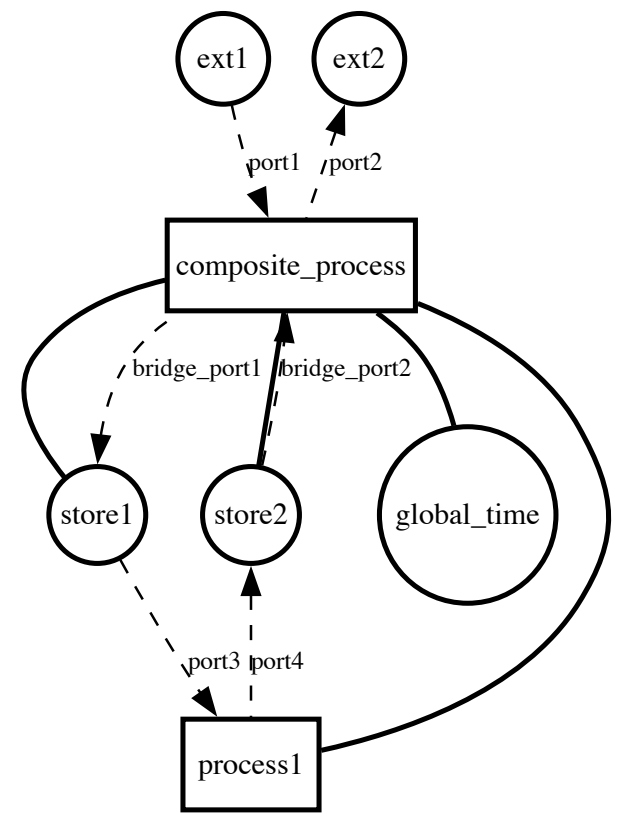

Figure S2: **State tree and diagram of a composite process.** *Left:* a state tree instantiating the declared ports and connecting them to stores. *Right:* the resulting process bigraph visualized with `bigraph-viz`.

3.8 Orchestration

The orchestration of processes draws on principles from discrete-event co-simulation, particularly the Discrete Event System Specification (DEVS) formalism [**zeigler2000theory**]. In this approach, processes are scheduled as events that operate on a shared state, enabling coordination between continuous-time dynamics and discrete updates. The orchestration layer determines execution order, time advancement, and synchronization, while ensuring that all processes observe a consistent system state.

In our setting, these ideas are reinterpreted for typed process bigraphs: simulation components become process nodes with typed ports that declare their own time advance, transitions become type-directed updates over schema/state trees, and a global orchestrator repeatedly executes the next imminent event.

3.8.1 Event times and schedule

Each process node $p \in \mathsf{LinkPath}$ carries:

- an *interval* value $\mathsf{interval}(p)$ declaring its nominal time advance;
- an *address* linking it to a handler class in the `link_registry` R_L (whose configured instance is stored at p in the state tree);
- typed input and output ports whose wiring is determined by the `inputs`/`outputs` entries in the state tree.

At runtime, the composite maintains:

- a global time $t \in \mathbb{R}_{\geq 0}$,
- a schedule $t_{\text{next}} : \mathsf{LinkPath} \to \mathbb{R}_{\geq 0}$ assigning each process node its next event time.

Initially, one may set $t_{\text{next}}(p) = t_0 + \mathsf{interval}(p)$ for an initial time t_0.

3.8.2 Small-step orchestration semantics

A configuration is a triple

$$\langle B, t, t_{\text{next}} \rangle,$$

where $B = (\Sigma, x, R_T, R_L)$ is a well-typed process bigraph, t is the current time, and t_{next} is the current schedule.

We define a transition

$$\langle B, t, t_{\text{next}} \rangle \longrightarrow \langle B', t', t'_{\text{next}} \rangle$$

by the following rules.

(Select) Scheduler step. Let

$$t^\star = \min_{p \in \mathsf{LinkPath}} t_{\text{next}}(p)$$

be the next event time. Let

$$S = \{\, p \in \mathsf{LinkPath} \mid t_{\text{next}}(p) = t^\star \,\}$$

be the set of all processes whose events occur at $t^\star$. We advance the global time to $t' = t^\star$. All processes in S then undergo a *Process Update*, conceptually in parallel when their deltas commute.

(Process Update). For each $p \in S$ — all processes updating at the same event time $t^\star$ — the runtime performs:

1. Perform input projection by reading the state paths listed in the `inputs` field at p, yielding x_{in}.
2. Invoke the configured handler instance stored at path p:

$$\delta_p = \mathsf{update}_p(x_{\text{in}},\, \Delta t),$$

   where $\Delta t = t^\star - t$. The result δ_p is a (possibly composite) delta structured by the output ports of p.
3. Apply δ_p to the state paths listed in the `outputs` field of p using the type engine's apply_τ operators.

Collectively applying all deltas $\{\delta_p \mid p \in S\}$ yields the updated state tree x' and schema tree Σ'. This produces the updated process bigraph $B' = (\Sigma', x', R_T, R_L)$, with internal wiring re-derived from the updated `inputs`/`outputs` fields.

(Reschedule). For each process node p that persists in B', set

$$t'_{\text{next}}(p) = t' + \mathsf{interval}(p),$$

unless overridden by process-specific semantics (e.g. disabling or pausing a process). Process nodes created or removed by structural deltas update the domain of t'_{next} accordingly.

3.8.3 Update reconciliation

When multiple processes produce updates to the same store during a single orchestration step, the resulting deltas must be reconciled before application. For many primitive numerical types this is straightforward because the update operation is commutative. For example, additive scalar or array deltas may simply be summed before application.

The challenge arises for non-commutative updates, where the order of application affects the result. Structural deltas are a common example: multiple updates may request subtree insertions, deletions, rewrites, or rewiring operations whose effects depend on execution order. Applying a deletion before a rewrite, for instance, may invalidate the target of another update.

To address this, types may define a *reconciler* associated with their update semantics, which takes a collection of deltas $(\delta_1, \delta_2, \ldots, \delta_n)$ and produces a single reconciled delta $\delta^\star$ that can then be applied through the standard type-directed operator $x' = \mathsf{apply}_\tau(x, \delta^\star)$.

Reconcilers separate how concurrent updates are merged from how the resulting update modifies the underlying value. For structural updates, a reconciler may impose a canonical ordering over edit operations, for example applying insertions first, then modifications, and finally deletions. This ensures deterministic structural transformations even when updates depend on one another. Because reconcilers are attached to types through the type registry R_T, different store types may implement different conflict-resolution semantics within the same orchestration framework.

3.8.4 DAG-structured discrete workflows

If all processes in a subsystem satisfy $\mathsf{interval}(p) = 0$, scheduling reduces to a directed acyclic graph (DAG) evaluation over these `step` nodes, with edges determined by data dependencies in the wiring.

Within a single global time t, execution proceeds as follows:

- a step p becomes *eligible* when all input ports updated in this tick have received new values;
- inputs unchanged in this tick do not block execution, allowing partially updated data to propagate;
- execution proceeds in topological order: once a step applies its output deltas, any successor steps whose inputs changed become eligible.

Cycles are disallowed, ensuring deterministic zero-time DAG evaluation within the broader event-scheduling semantics.

Because `Composite` nodes can behave like ordinary processes, an entire composite simulation can itself appear as a single `step` in a larger DAG. This enables end-to-end workflows in which multiple simulations and other tools such as parameter estimation and analysis can be integrated within the same framework.

3.8.5 Recursive composition

Because a `Composite` encapsulates its own scheduler, stores, and internal processes, it acts externally as a process but internally executes a complete process bigraph. For temporal processes inside the composite, the internal scheduler maintains its own event queue, advancing the front of that schedule in time and updating the embedded state and schema as structural deltas create, remove, or move process nodes. The composite therefore continually re-tracks all available internal processes, their paths, and their next update times. After each internal update, the composite advances its own event time according to its interval field, and outward-facing results are propagated through its interface ports via the `bridge`. In this way, temporal dynamics, DAG-style workflows, and structural graph rewrites coexist naturally within a single hierarchical system under uniform schema-based, typed-link semantics.

3.8.6 Large-scale orchestration

The orchestration semantics above describe the logical execution model of a process bigraph. At runtime, large simulations may involve many processes executing through heterogeneous protocols, including local execution, multiprocessing, distributed Ray actors, or remote REST services. To support this, the engine organizes execution into a staged orchestration pipeline that separates scheduling, invocation, synchronization, and application.

A call to `run`(Δt) advances the simulation by repeatedly invoking the following pipeline until the requested interval has elapsed:

1. **Partition.** Scheduled processes are first partitioned by execution protocol. Local processes execute directly, while distributed protocols such as Ray or REST may batch or collate calls for remote execution. This allows each protocol backend to manage serialization, communication, and concurrency independently.
2. **Invoke.** Each process invocation proceeds through four substeps:
   (i) *Slice*: project the current state tree onto the paths referenced by the process's wired input ports;
   (ii) *Interval*: compute the timestep via calculate_timestep, typically returning the configured `interval`;
   (iii) *Invoke*: call the process handler with the projected inputs and timestep, producing a typed update delta, potentially asynchronously for distributed protocols;
   (iv) *Stash*: store the returned delta in the engine's execution front together with the simulated time reached by the process.
3. **Flush.** After asynchronous invocations complete, the engine collates all returned deltas into a unified update collection.
4. **Apply.** Application then proceeds through three substeps:
   (i) *Combine*: gather all updates targeting the same store path;
   (ii) *Reconcile*: resolve non-commutative updates through the type-associated reconciler described in Section 3.8.3;
   (iii) *Apply*: apply the reconciled update using the corresponding type-directed operator apply_τ.

3.9 Emitters and result capture

Emitters are special `step` processes whose role is observational of the simulation state for saving results. An emitter declares typed input ports, but no semantic outputs back into the simulation state. At execution time, the emitter reads values from its input ports and records them into an external or side-channel store for later retrieval, analysis, or visualization.

Operationally, an emitter participates in orchestration exactly like any other step process: when scheduled, it performs input projection, invokes its handler, and produces a delta. However, the delta produced by an emitter is it writing to a recording backend (e.g. in-memory buffers, disk, streaming logs), rather than as an update to the schema/state tree. Emitters therefore do not introduce new data dependencies into the process bigraph and cannot affect subsequent process execution.

3.10 Protocols

Process-bigraph protocols define mechanisms for resolving process addresses and executing process updates under different runtime and communication models.

Local execution. Local process resolution is handled through `bigraph_schema.protocols.local_lookup_module`. In this mode, processes are instantiated directly as Python objects within the runtime, and updates are computed via in-memory function calls without serialization or inter-process communication.

Parallel execution. The `parallel` protocol executes each process in a separate operating-system process using Python `multiprocessing`. Inter-process communication is implemented via `multiprocessing.Pipe`, with serialized state exchanged between the main scheduler and worker processes. This protocol provides true parallel execution on a single node while maintaining a shared logical state.

REST execution. The `rest` protocol maps process updates to HTTP requests against a remote service. A process address resolves to a tuple {`process, host, port`}, and each update step is executed through `GET` or `POST` requests. State is serialized into request payloads and deserialized from responses, enabling integration with externally hosted models and services.

Ray execution. The `ray` protocol implements distributed execution using Ray actors. Each unique `(process_class, config)` pair is associated with a persistent pool of actors (default size `os.cpu_count()`). Process update calls are dispatched to actors in a round-robin manner, ensuring bounded memory usage independent of the number of process clients. Actor pools persist across `Composite` executions and support concurrent scheduling when using `parallel_processes=True`.

Extensions and prior protocol variants. A previous `docker` protocol provided container-based execution by wrapping processes in Docker containers. Although this implementation is not part of the current protocol registry, containerized execution remains a natural extension for integrating compiled simulators, command-line tools, and dependency-isolated model services. A `socket` protocol has also been explored as a lower-level communication interface for process execution, but is not currently exposed as an active runtime protocol.
