# Supplementary material for: Process bigraphs and the architecture of compositional systems biology
Source: PLoS Comput Biol. 2026 Oct 9;22(10):e1014819. doi: 10.1371/journal.pcbi.1014819 (PMC13652326; doi:10.1371/journal.pcbi.1014819)
Supplement: Supplementary file 2 [file pcbi.1014819.s002.pdf]

Supplement #2 for

# Process Bigraphs and the Architecture of Compositional Systems Biology

Eran Agmon *

Ryan K. Spangler

Center for Cell Analysis and Modeling,
University of Connecticut Health,
Farmington, Connecticut, USA

August 13, 2026

## Contents

**1 Spatio-Flux Instantiation** **1**
- 1.1 Spatio-Flux types . . . 1
  - 1.1.1 Primitive scientific types . . . 2
  - 1.1.2 Concentration/count coupled types . . . 2
  - 1.1.3 Particle record types . . . 3
  - 1.1.4 Structured store types . . . 4
- 1.2 Spatio-Flux processes . . . 4
  - 1.2.1 Local metabolic processes . . . 5
  - 1.2.2 Field movement . . . 7
  - 1.2.3 Particle movement processes . . . 8
  - 1.2.4 Particle–field adapters . . . 11
  - 1.2.5 Structural and Boundary – Graph rewrites . . . 12
- 1.3 Particle–COMETS reference composite . . . 15

**2 Performance and comparison** **22**
- 2.1 Performance . . . 22
- 2.2 Comparison with COMETS . . . 25

*Correspondence: agmon@uchc.edu

# 1 Spatio-Flux Instantiation

This section gives a concrete example of the Process–Bigraph applied to the Spatio-Flux example from the main text. We begin by introducing some of Spatio-Flux's processes in 1.2. These are then integrated for the reference demonstration in 1.3, with all the required config parameters and wiring. All documents and tests for this example are available in the public `spatio-flux` repository, as well as many other novel compositions.

The repository also contains an automated test suite that serves as a living catalog of compositional patterns. Tests are defined as executable composite documents and are designed to be extended as new process families and adapters are added. Because the suite is intended to evolve alongside the library, individual test configurations may be refined over time while preserving their role as reproducible examples and semantic checks.

## 1.1 Spatio-Flux types

Spatio-Flux instantiates the Process–Bigraph type system through a small collection of reusable scientific and structural types. These types define the semantic contracts for spatial fields, particles, concentrations, exchange fluxes, and mechanical attributes, while also specifying how updates are validated, reconciled, and applied. At runtime, these types appear as the schemas governing the `fields`, `particles`, and auxiliary stores used throughout the Spatio-Flux composites.

Table 1 summarizes the major type families used by Spatio-Flux. The types progress from low-level scalar and concentration representations, to particle records and mechanical extensions, and finally to the large structured stores that organize the simulation state. Together, they define the semantic substrate through which all Spatio-Flux processes communicate.

| Type family | Types |
|---|---|
| Primitive scientific | `concentration`, `counts`, `position`, `velocity` |
| Concentration/count coupled | `conc_counts` |
| Particle records | `particle`, `complex_particle` |
| Structured stores | `fields`, `particles`, `global_time` |

Table 1: **Spatio-Flux type families.** Each family links to a detailed subsection describing its semantics and update behavior.

### 1.1.1 Primitive scientific types

Primitive Spatio-Flux types represent the basic physical quantities exchanged between processes. These include scalar concentrations, molecular counts, particle positions, and velocities. Most primitive numerical types use additive update semantics, where emitted deltas are summed and then applied directly to the stored value.

**Primitive scientific types** primitive

The primitive scientific types provide the basic numerical substrate used throughout the Spatio-Flux schemas and state trees.

| type primitive | |
|---|---|
| **Field** | **Type** |
| concentration | `float`; scalar concentration value |
| counts | `float`; molecular count or amount |
| position | `tuple[float,float]`; spatial coordinates |
| velocity | `tuple[float,float]`; velocity vector |

**Semantics and updates.** Scalar numerical types use additive updates

$$x \leftarrow x + \Delta,$$

while tuple-valued quantities apply updates element-wise. These primitive types form the leaves of larger structured particle and field records.

### 1.1.2 Concentration/count coupled types

The `conc_counts` type couples concentration and molecular-count representations into a single typed object. This allows field processes and metabolic processes to operate simultaneously on concentration-derived dynamics and count-conserving updates while maintaining consistency between the two representations.

**Coupled concentration/count representation** field site

| type conc_counts | |
|---|---|
| **Field** | **Type** |
| concentration | `float`; derived concentration value |
| counts | `float`; conserved molecular amount |
| volume | `float`; local site volume |

**Semantics and updates.** The `counts` field is treated as the primary conserved quantity, while concentration is maintained as the invariant

$$c = \frac{n}{V}.$$

Given a count delta $\Delta n$, the type-specific update operator computes

$$\text{apply}_{\text{conc\_counts}}((c, n), \Delta n) = \left( \frac{n + \Delta n}{V}, n + \Delta n \right).$$

This allows metabolic processes to emit local count deltas while field processes consume concentration-derived quantities.

### 1.1.3 Particle record types

Particle types represent mobile biological agents embedded within the spatial environment. Each particle is stored as a structured record containing biological, spatial, and optionally mechanical state. Processes interact with particles through typed map updates keyed by particle identifier.

**Particle** particle agent

| type particle | |
|---|---|
| **Field** | **Type** |
| `id` | `string`; globally unique particle identifier |
| `position` | `position`; spatial coordinates |
| `mass` | `concentration`; particle biomass |
| `local` | `map[concentration]`; local substrate state |
| `exchange` | `map[counts]`; exchange deltas with fields |

**Semantics and updates.** Particle records are updated field-wise through recursive map semantics. Movement processes typically update `position`, metabolic processes modify `mass` and `exchange`, and structural processes may emit `_add` or `_remove` deltas on the enclosing particle map.

**Complex particle** mechanical particle agent

| type complex_particle | |
|---|---|
| **Field** | **Type** |
| `_inherit` | `particle`; inherits base particle schema |
| `velocity` | `velocity`; particle velocity |
| `radius` | `float`; collision radius |
| `friction` | `float`; contact friction coefficient |
| `elasticity` | `float`; collision elasticity |
| `inertia` | `float`; rigid-body inertia |

**Semantics and updates.** The `complex_particle` type extends the base particle schema with explicit mechanical state used by Newtonian particle dynamics and rigid-body simulations. Movement processes update position and velocity continuously, while collision and boundary events may trigger structural rewrites of the enclosing particle map.

### 1.1.4 Structured store types

The top-level Spatio-Flux stores organize particles, fields, and global simulation state into typed hierarchical structures. These stores form the shared substrate through which all processes communicate.

**Top-level simulation stores** structured stores

| type stores | |
|---|---|
| **Field** | **Type** |
| fields | map[array[conc_counts]]; spatial substrate lattices |
| particles | map[complex_particle]; particle population |
| global_time | float; simulation clock |

**Semantics and updates.** The `fields` store maps each substrate identifier to a two-dimensional lattice of `conc_counts` sites. The `particles` store is a keyed map from particle identifiers to particle records and supports structural updates through `_add` and `_remove` deltas. Array-valued stores support both dense array updates and sparse indexed updates targeting selected lattice positions. Together, these stores provide the shared typed state used by all Spatio-Flux processes.

### 1.2 Spatio-Flux processes

Spatio-Flux instantiates the Process Bigraph semantics through a small set of reusable processes that can be wired into the `fields` and/or `particles` stores. Each process is a `process` or `step` node whose schema specifies typed input and output ports and whose state-tree entry stores its `address`, `config`, and wiring to concrete store paths. Upon execution, each process receives an input projection, computes an update delta tree, and the orchestration engine applies it.

Table 2 summarizes the main process families, organized by modeling concern and linked to the corresponding subsections below. These families progress from local metabolic dynamics, to spatial transport, to particle motion, to adapter processes that couple representations, and finally to structural graph rewrites that modify population topology. The detailed process "cards" that follow provide a concrete reference for each process's interface, configuration, and semantics.

The process cards were generated semi-automatically from process schemas, docstrings, and reference configurations, with light manual editing to improve presentation. A more streamlined card-generation pipeline may be integrated into `spatio-flux` in future releases, enabling documentation and test-suite examples to be rendered from the same source artifacts.

| **Process family** | **Processes** |
|---|---|
| Metabolic | `DynamicFBA`, `MonodKinetics`, `SpatialDFBA` |
| Field transport | `DiffusionAdvection` |
| Particle movement | `BrownianMovement`, `PymunkParticleMovement` |
| Particle–field adapters | `ParticleExchange`, `ParticleTotalMass` |
| Structural and boundary | `ParticleDivision`, `ManageBoundaries` |

Table 2: **Spatio-Flux process families.** Each family links to a detailed subsection describing its processes and semantics.

### 1.2.1 Local metabolic processes

Metabolic processes operate on individual lattice sites, which store derived concentrations. Each metabolic process reads local substrate concentrations and biomass, computes reaction rates, and writes back typed count deltas to the same site, ensuring compatibility with the `conc_counts` update semantics. Because the metabolic processes share a common typed interface, they can be interchanged freely at any lattice location.

**Monod kinetics (ODE)** local metabolic

The `monod_kinetics` process provides a saturating-rate ODE for uptake and secretion at a single lattice site. It implements the `metabolism` interface with a compact configuration in terms of reactions and kinetic parameters.

**Interface**

`process monod_kinetics`

| **Name** | **Direction** | **Type** |
|---|---|---|
| biomass | input | concentration |
| substrates | input | map[concentration] |
| biomass | output | counts |
| substrates | output | map[counts] |

**Parameters**

| **Parameter** | **Type** | **Default** | **Role** |
|---|---|---|---|
| reactions | map[reaction] | {`assimilate_glucose`, `maintenance_turnover`} | Reaction definitions mapping `reactant` and `product` species for each Monod term. |
| kinetic_params | map[tuple[float,float]] | `glucose` : $(0.5, 0.01)$; `mass` : $(1.0, 0.001)$ | Per-species Monod parameters $(K_m, V_{\max})$ for substrate uptake and mass turnover. |

**Semantics and equations.** For each configured Monod term with reactant $R$ (a substrate concentration $c_R$, or biomass $m$ if $R = \texttt{mass}$) and product $P$, the site-local rate is

$$v_R \;=\; \frac{V_{\max,R}\, c_R}{K_{m,R} + c_R}.$$

Over timestep $\Delta t$, the process returns stoichiometric deltas in `counts` form: reactant is consumed $\Delta N_R = -v_R \Delta t$, biomass increases when $P = \texttt{mass}$ via $\Delta m = v_R \Delta t$, and any product substrate $S$ receives $\Delta N_S = +v_R \Delta t$.

**Dynamic FBA (network-level metabolism)** local metabolic

The `dfba` process uses a full metabolic network model and computes feasible instantaneous fluxes using `cobraPy`. It implements the same `metabolism` interface as `monod_kinetics`, but with a configuration that points to a COBRA model and specifies how substrates are mapped to exchange reactions.

**Interface**

| process dfba | | |
|---|---|---|
| **Name** | **Direction** | **Type** |
| biomass | input | concentration |
| substrates | input | map[concentration] |
| biomass | output | counts |
| substrates | output | map[counts] |

**Parameters**

| Parameter | Type | Default | Role |
|---|---|---|---|
| model_file | string | required | Path to the COBRA metabolic model used for FBA. |
| kinetic_params | map[tuple[float,float]] | required | Per-substrate Michaelis–Menten parameters $(K_m, V_{\max})$ used to set uptake bounds. |
| substrate_update_reactions | map[string] | required | Map from substrate identifiers to exchange reaction IDs in the model. |
| bounds | map[bounds] | required | Optional overrides for exchange-reaction lower/upper bounds. |

**Semantics and equations.** For each substrate $s$ with concentration $c_s$, an uptake bound is computed via Michaelis–Menten kinetics and applied to the corresponding exchange reaction:

$$v_s^{\text{uptake}} \;=\; -\frac{V_{\max,s}\, c_s}{K_{m,s} + c_s}.$$

Given bounds $\ell \leq v \leq u$, the COBRA model solves an instantaneous FBA problem (typically maximizing growth), yielding an optimal growth rate $\mu$ and exchange fluxes $v_s$. The returned `counts`-typed deltas over $\Delta t$ are

$$\Delta m \;=\; \mu\, m\, \Delta t, \qquad \Delta N_s \;=\; v_s\, m\, \Delta t,$$

with substrate deltas clamped to preserve non-negativity under `conc_counts` semantics.

### 1.2.2 Field movement

Field movement processes operate on the two-dimensional arrays of typed `conc_counts` sites stored in the `fields` map. Each species occupies a lattice of concentrations, and the field processes update these concentrations by computing spatial fluxes and returning typed deltas that the `conc_counts_apply` operator converts into consistent `counts` and `concentration` updates. Because the interface is typed, field processes integrate seamlessly into the Process–Bigraph semantics: inputs project each species array, the handler computes spatial fluxes, and outputs return per-site deltas under the `fields` port.

**Diffusion–advection** field movement

The `diffusion` process updates the concentration fields of all substrates on the spatial lattice using an explicit advection–diffusion scheme. It reads the current concentration arrays for each species and returns typed deltas that modify the same arrays in place.

**Interface**

`process diffusion`

| **Name** | **Direction** | **Type** |
|---|---|---|
| fields | input | map[array[concentration]] |
| fields | output | map[array[concentration]] |

**Parameters**

| Parameter | Type | Default | Role |
|---|---|---|---|
| n_bins | tuple[integer,integer] | required | Number of lattice cells in $(x, y)$. |
| bounds | tuple[float,float] | required | Physical domain size in $(L_x, L_y)$. |
| default_diffusion _rate | float | `1e-1` | Default diffusion coefficient $D$ used if no species-specific value is given. |
| diffusion_coeffs | map[float] | none | Per-species diffusion coefficients overriding the default. |
| advection_coeffs | map[tuple[float,float]] | none | Per-species advection velocities $(v_x, v_y)$. |
| max_dt | float | `1e-1` | Upper bound on internal diffusion timestep. |
| cfl_adv | float | `0.5` | CFL stability factor for the advection term. |
| clip_negative | boolean | `true` | If true, negative concentrations are clipped to zero. |

**Semantics and equations.** For each species field $C_s$, concentrations evolve according to

$$\partial_t C_s \;=\; D_s \nabla^2 C_s \;-\; \mathbf{v}_s \cdot \nabla C_s.$$

The handler selects a stable internal timestep $\delta t$ (CFL-limited and capped by `max_dt`) and advances the field with $n = \lceil \Delta t / \delta t \rceil$ explicit Euler substeps using standard finite-difference stencils. The emitted output is a per-species delta array

$$\Delta C_s = C_s^{\text{final}} - C_s^{\text{initial}},$$

optionally clipped to enforce non-negative concentrations.

### 1.2.3 Particle movement processes

Particle movement processes operate on the `particles` store, a typed map from particle identifiers to particle records. The schema distinguishes base `particle` records from `complex_particle` records that also include mechanical attributes (shape, velocity, inertia, friction, elasticity, radius). Each movement process is a typed `process` node whose ports are wired to `particles` and whose update method returns deltas that modify particle attributes and, when needed, perform structural rewrites on the particle map.

**Brownian movement** particle movement

The `brownian_movement` process models overdamped drift–diffusion of particles inside a rectangular domain. It acts on base `particle` records and updates only the `position` field of

each surviving particle, with optional boundary birth/death.

**Interface**

| process brownian_movement | | |
|---|---|---|
| **Name** | **Direction** | **Type** |
| particles | input | map[particle] |
| particles | output | map[particle] |

**Parameters**

| Parameter | Type | Default | Role |
|---|---|---|---|
| bounds | tuple[float,float] | required | Spatial domain size used to clamp particle positions. |
| diffusion_rate | float | `1e-1` | Brownian diffusion coefficient $D$. |
| advection_rate | tuple[float,float] | (0.0, 0.0) | Uniform drift velocity $(v_x, v_y)$. |

**Semantics and equations.** Each particle position $X_i \in \mathbb{R}^2$ is updated over $\Delta t$ using an Euler–Maruyama drift–diffusion step:

$$X_i \leftarrow X_i + \mathbf{v}\,\Delta t + \sqrt{2D\,\Delta t}\,\xi_i, \qquad \xi_i \sim \mathcal{N}(0, I_2).$$

The process emits typed deltas that update only the `position` field of each particle. Particles that cross configured absorbing boundaries may be removed via structural `_remove` deltas when boundary-driven rewrites are enabled; otherwise, positions are unconstrained by the process itself.

**Newtonian particles** particle movement

The `newtonian_particles` process refines particle mechanics by treating each particle as a rigid body in a 2D physics engine (Pymunk). It acts on `complex_particle` records with explicit mechanical attributes and updates both positions and velocities, with optional boundary birth/death.

**Interface**

| process newtonian_particles | | |
|---|---|---|
| **Name** | **Direction** | **Type** |
| particles | input | map[particle] |
| particles | output | map[particle] |

**Parameters**

| Parameter | Type | Default | Role |
|---|---|---|---|
| bounds | tuple[float,float] | (500.0, 500.0) | Physical domain size for particle motion. |
| diffusion_rate | float | `1e-1` | Effective mechanical diffusion strength (jitter scale). |
| advection_rate | tuple[float,float] | (0.0, 0.0) | Global drift velocity applied to all particles. |
| substeps | integer | `100` | Number of Pymunk substeps per external timestep. |
| damping_per _second | float | `0.98` | Global velocity damping per second. |
| gravity | float | `-9.81` | Vertical gravitational acceleration. |
| friction | float | `0.8` | Contact friction coefficient. |
| elasticity | float | `0.0` | Contact elasticity (bounciness). |
| jitter_per_second | float | `1e-2` | Standard deviation of random impulse per second. |
| barriers | list[map] | `[]` | Optional internal obstacles/walls definitions. |
| wall_thickness | float | `100.0` | Thickness of outer domain walls. |

**Semantics and equations.** Particles are simulated as rigid bodies governed by Newtonian mechanics,

$$m\,\frac{dV}{dt} = F_{\text{net}}, \qquad \frac{dX}{dt} = V,$$

implemented via a 2D physics engine with configured gravity, damping, contact mechanics, and stochastic jitter. Over an external timestep $\Delta t$, the engine advances in multiple substeps, after which the process emits deltas updating particle positions, velocities, and other mechanical attributes. Boundary crossings may additionally trigger `_add`/`_remove` rewrites on the particle map.

#### 1.2.4 Particle–field adapters

**Particle–field exchange** adapter

`particle_exchange` is a `step` node that acts as an adapter between the continuous particle representation and the discrete field lattice. It introduces no new physics of its own; instead, it (i) samples lattice concentrations at particle locations into per-particle `local` state, and (ii) applies per-particle `exchange` *amounts* into the appropriate lattice bins as *concentration* updates while preserving mass balance by converting amounts to concentration deltas using

the bin volume implied by the spatial discretization.

**Interface**

| step particle_exchange | | |
|---|---|---|
| **Name** | **Direction** | **Type** |
| particles | input | map[particle] |
| fields | input | fields |
| particles | output | map[particle] |
| fields | output | fields |

**Parameters**

| **Parameter** | **Type** | **Default** | **Role** |
|---|---|---|---|
| bounds | tuple[float,float] | required | Domain bounds $(L_x, L_y)$ used to map particle positions to lattice indices. |
| n_bins | tuple[integer,integer] | required | Number of lattice cells $(n_x, n_y)$; must match the `fields` grid. |
| depth | float | required | Assumed depth of the 2D lattice used to compute per-bin volume. |

**Semantics and equations.** Each particle position $X_i = (x_i, y_i)$ is mapped to a lattice cell $(c_i, r_i)$ by uniform binning over $[0, L_x] \times [0, L_y]$. Let $C_m(c, r)$ denote the field concentration of species $m$ at that cell. The adapter updates each particle's `local` snapshot by sampling:

$$\texttt{local}_{i,m} \leftarrow C_m(c_i, r_i),$$

emitting either an initial `_add` (first sample) or a delta update thereafter.

To preserve mass balance between particle-local `exchange` *amounts* and field *concentrations*, the adapter converts each queued per-particle exchange amount $\Delta A_{i,m}$ into a concentration delta in the target bin using the bin volume

$$V_{\text{bin}} \;=\; \Delta x\, \Delta y\, \text{depth}, \qquad \Delta x = \frac{L_x}{n_x},\ \Delta y = \frac{L_y}{n_y}.$$

For each species $m$, it applies

$$\Delta C_m(c_i, r_i) \mathrel{+}= \frac{\Delta A_{i,m}}{V_{\text{bin}}}.$$

Finally, consumed exchange entries are reset to zero via particle deltas, ensuring one-shot transfer semantics.

**Aggregate particle mass** adapter

The `aggregate_mass` process is a `step` node that acts as an adapter between compositional internal particle state and a single scalar mass used by downstream processes. It introduces no new dynamics of its own; instead, it aggregates multiple per-particle `sub_masses` contributions into a single `total_mass` value, providing a well-defined interface between internal metabolic structure and particle-level structural rules.

**Interface**

| step aggregate_mass | | |
|---|---|---|
| **Name** | **Direction** | **Type** |
| sub_masses | input | map[mass] |
| total_mass | output | overwrite[mass] |

**Parameters**

| **Parameter** | **Type** | **Default** | **Role** |
|---|---|---|---|

**Semantics and equations.** Let $\{ m_k \}_{k\in K}$ denote the values stored in the particle-local `sub_masses` map. The adapter computes the total particle mass as a simple aggregation,

$$m_{\text{total}} = \sum_{k\in K} m_k,$$

ignoring any non-numeric entries defensively. The resulting scalar is emitted as an `overwrite` update to `total_mass`, replacing any previous value. By separating internal mass accumulation from its downstream interpretation, this adapter allows particle-level structural processes (e.g. division, removal, or mechanical scaling) to depend on a single scalar signal while preserving compositional internal structure.

#### 1.2.5 Structural and Boundary – Graph rewrites

**Particle division** graph rewrite

The `particle_division` process is a `step` node whose primary role is to demonstrate graph rewrites on the `particles` store. It has a purely particle-based interface and performs no continuous time integration; instead, it rewrites the particle map whenever a mass threshold is exceeded.

**Interface**

| step particle_division | | |
|---|---|---|
| **Name** | **Direction** | **Type** |
| particles | input | map[particle] |
| particles | output | map[particle] |

**Parameters**

| Parameter | Type | Default | Role |
|---|---|---|---|
| division_mass _threshold | float | `0.0` | Mass threshold above which particles are eligible to divide (disabled if $\leq 0$). |
| division_jitter | float | `1e-3` | Fraction of a reference length used as jitter radius to place daughter particles. |

**Semantics and equations.** For each particle with mass $m_i \geq m_{\text{thr}}$, the process performs a graph rewrite: the parent particle is removed and two daughters are inserted. Daughter positions are displaced symmetrically by a small random offset, and masses split the parent mass,

$$m_{i,1} = m_{i,2} = \tfrac{1}{2} m_i.$$

All changes are emitted as `_remove` and `_add` deltas on the `particles` map.

**Manage boundaries (reflect & absorb)** boundary policy

The `manage_boundaries` step enforces boundary policies on particle *absolute* positions. It reads the `particles` map, applies absorbing removal for configured sides, reflects all other out-of-range coordinates back into the domain interior, and can spawn new particles at selected boundaries according to a Poisson birth process.

**Interface**

| step manage_boundaries | | |
|---|---|---|
| **Name** | **Direction** | **Type** |
| particles | input | map[particle] |
| process_interval | input | float |
| particles | output | map[particle] |

**Parameters**

| Parameter | Type | Default | Role |
|---|---|---|---|
| bounds | tuple[float,float] | required | Domain size $(L_x, L_y)$ defining hard bounds $[0, L_x] \times [0, L_y]$. |
| add_rate | float | `0.0` | Birth event rate (1/sec) used to sample boundary-spawn events. |
| boundary_to_add | list[boundary_side] | `["top"]` | Sides where new particles may be spawned. |
| boundary_to _remove | list[boundary_side] | `[]` | Absorbing sides; particles crossing a configured side are removed. |
| buffer | float | `1e-6` | Numerical buffer used to reflect into the interior to avoid floating-point sticking on walls. |
| mass_range | tuple[float,float] | `INITIAL_MASS_RANGE` | Mass range used when generating newborn particles. |

**Semantics and equations.** Let the hard domain be $[0, L_x] \times [0, L_y]$ and the interior reflection domain be $[\,\epsilon,\ L_x - \epsilon\,] \times [\,\epsilon,\ L_y - \epsilon\,]$ with $\epsilon =$ `buffer`. For each particle id $i$ with absolute position $X_i = (x_i, y_i)$:

1. *Absorbing removal.* If $X_i$ violates a configured absorbing side (e.g. $x_i < 0$ when `left` is absorbing, $x_i > L_x$ when `right` is absorbing, etc.), emit a structural `_remove` delta for $i$.
2. *Reflection.* Otherwise, any out-of-range coordinate is reflected back into the interior interval. In one dimension, reflection into $[lo, hi]$ is computed by

$$\text{reflect}(x; lo, hi) \ = \ \begin{cases} lo + y, & y \le (hi - lo) \\ hi - (y - (hi - lo)), & y > (hi - lo) \end{cases} \qquad y = (x - lo) \bmod 2(hi - lo),$$

   and applied componentwise:

$$(x_i', y_i') = \big(\text{reflect}(x_i; \epsilon, L_x - \epsilon),\ \text{reflect}(y_i; \epsilon, L_y - \epsilon)\big).$$

   Emit an *absolute* position update delta for particle $i$: $\Delta_i = \{\texttt{position} : (x_i', y_i')\}$.

*Boundary births.* Let $\Delta t =$ `process_interval` and birth rate $\lambda =$ `add_rate`. For each boundary $b \in$ `boundary_to_add`, sample an event with probability

$$p_{\text{birth}} = 1 - e^{-\lambda \Delta t}.$$

On success, generate a new id $j$, choose an absolute spawn position $X_j$ on side $b$, sample mass from `mass_range`, and emit a structural `_add` delta inserting the new particle record.

The output under `particles` therefore combines (i) attribute deltas for absolute position updates, and (ii) structural edits `_remove`/`_add` implementing absorbing boundaries and Poisson births.

### 1.3 Particle–COMETS reference composite

This subsection lists the concrete wiring and parameter values used for the reference composite shown in the main text, instantiated as a single `Composite`. The `spatio-flux` test suite includes a designated reference composite for regression and demonstration, but its exact configuration may evolve as new processes become available and improved models and parameters are integrated. At the time of writing these were the configuration fields and values.

`composite particle_comets`

**diffusion** field transport

`process diffusion`

**Wiring**

| Port | Direction | Store path |
|---|---|---|
| `fields` | input | `fields` |
| `fields` | output | `fields` |

**Properties**

| Property | Value |
|---|---|
| address | `local.DiffusionAdvection` |
| interval | `1.0` |

**Config**

| Key | Value |
|---|---|
| bounds | [50.0, 50.0] |
| n_bins | [10, 10] |
| default_diffusion_rate | 0.1 |
| default_diffusion_dt | 0.1 |
| max_dt | 0.1 |
| cfl_adv | 0.5 |
| clip_negative | true |
| diffusion_coeffs.glucose | 0.1 |
| diffusion_coeffs.acetate | 0.1 |
| diffusion_coeffs.dissolved biomass | 0.1 |
| advection_coeffs.glucose | [0.0, 0.0] |
| advection_coeffs.acetate | [0.0, 0.0] |
| advection_coeffs.dissolved biomass | [0.0, 0.0] |
| boundary_conditions.default.x.type | periodic |
| boundary_conditions.default.y.type | neumann |
| boundary_conditions.glucose.top.type | dirichlet |
| boundary_conditions.glucose.top.value | 5.0 |
| boundary_conditions.acetate.bottom.type | dirichlet |
| boundary_conditions.acetate.bottom.value | 5.0 |

composite particle_comets

**monod_kinetics[i,j]** local metabolic

process monod_kinetics

**Wiring**

| Port | Direction | Store path |
|---|---|---|
| substrates.glucose | input | fields.glucose[i,j] |
| substrates.acetate | input | fields.acetate[i,j] |
| biomass | input | fields.dissolved biomass[i,j] |
| substrates.glucose | output | fields.glucose[i,j] |
| substrates.acetate | output | fields.acetate[i,j] |
| biomass | output | fields.dissolved biomass[i,j] |

**Properties**

| Property | Value |
|---|---|
| address | local.MonodKinetics |
| interval | 1.0 |

**Config**

| Key | Value |
|---|---|
| reactions.assimilate_glucose.reactant | glucose |
| reactions.assimilate_glucose.product | mass |
| reactions.assimilate_glucose.km | 1.0 |
| reactions.assimilate_glucose.vmax | 0.02 |
| reactions.assimilate_glucose.yield | 0.15 |
| reactions.overflow_to_acetate.reactant | glucose |
| reactions.overflow_to_acetate.product | acetate |
| reactions.overflow_to_acetate.km | 1.0 |
| reactions.overflow_to_acetate.vmax | 0.2 |
| reactions.overflow_to_acetate.yield | 0.85 |
| reactions.maintenance_turnover.reactant | mass |
| reactions.maintenance_turnover.product | detritus |
| reactions.maintenance_turnover.km | 1.0 |
| reactions.maintenance_turnover.vmax | 0.001 |
| reactions.maintenance_turnover.yield | 1.0 |

composite particle_comets

**newtonian_particles** particle movement

process newtonian_particles

**Wiring**

| Port | Direction | Store path |
|---|---|---|
| particles | input | particles |
| particles | output | particles |

**Properties**

| Property | Value |
|---|---|
| address | local.PymunkParticleMovement |
| interval | 1.0 |

**Config**

| Key | Value |
|---|---|
| bounds | [50.0, 50.0] |
| substeps | 100 |
| gravity | -1.0 |
| damping_per_second | 0.95 |
| jitter_per_second | 0.01 |
| diffusion_rate | 0.1 |
| advection_rate | [0.0, 0.0] |
| friction | 0.9 |
| elasticity | 0.1 |
| wall_thickness | 100.0 |
| barriers | [] |
| new_particle_radius_range | [0.5, 2.0] |
| new_particle_mass_range | [1.0, 1.0] |
| new_particle_density | 0.015 |
| new_particle_speed_range | [0.0, 0.0] |
| circle_density | 0.015 |
| segment_radius | 10.0 |
| segment_density | 0.02 |
| segment_radius | 10.0 |

composite particle_comets

**particle_exchange**

particle–field coupling

step particle_exchange

### Wiring

| Port | Direction | Store path |
|---|---|---|
| particles | input | particles |
| fields | input | fields |
| particles | output | particles |
| fields | output | fields |

### Properties

| Property | Value |
|---|---|
| address | local.ParticleExchange |
| interval | 1.0 |

### Config

| Key | Value |
| --- | --- |
| bounds | [50.0, 50.0] |
| n_bins | [10, 10] |
| apply_mass_balance | false |
| rates_are_per_time | true |

composite particle_comets

**enforce_boundaries** boundary management

step enforce_boundaries

### Wiring

| Port | Direction | Store path |
| --- | --- | --- |
| particles | input | particles |
| process_interval | input | newtonian_particles.interval |
| particles | output | particles |

### Properties

| Property | Value |
| --- | --- |
| address | local.ManageBoundaries |
| interval | 1.0 |

### Config

| Key | Value |
| --- | --- |
| bounds | [50.0, 50.0] |
| buffer | 0.0001 |
| clamp_survivors | true |
| mass_range | [0.001, 1.0] |
| add_rate | 0.0 |
| boundary_to_remove | [] |
| boundary_to_add | ["top","top","bottom","left","right"] |

composite particle_comets

**particle_division** graph rewrite

step particle_division

### Wiring

| Port | Direction | Store path |
|---|---|---|
| particles | input | particles |
| particles | output | particles |

### Properties

| Property | Value |
|---|---|
| address | local.ParticleDivision |
| interval | 1.0 |

### Config

| Key | Value |
|---|---|
| division_mass_threshold | 0.4 |
| division_jitter | 0.001 |
| sub_mass_split | equal |
| submass_split_mode | random |

composite particle_internal

## ecoli_1 dFBA

particle metabolic

process ecoli_1 dFBA

### Wiring

| Port | Direction | Store path |
|---|---|---|
| substrates | input | local |
| biomass | input | sub_masses.ecoli_1 |
| substrates | output | exchange |
| biomass | output | sub_masses.ecoli_1 |

### Properties

| Property | Value |
|---|---|
| address | local.DynamicFBA |

### Config

| Key | Value |
|---|---|
| model_file | textbook |
| kinetic_params.glucose | [0.1, 2.0] |
| kinetic_params.acetate | [1.0, 0.1] |
| substrate_update_reactions.glucose | EX_glc__D_e |
| substrate_update_reactions.acetate | EX_ac_e |
| bounds.ATPM.lower | 1.0 |
| bounds.ATPM.upper | 1.0 |
| bounds.EX_o2_e.lower | -2.0 |

composite particle_internal

### ecoli_2 dFBA

particle metabolic

process ecoli_2 dFBA

#### Wiring

| Port | Direction | Store path |
|---|---|---|
| substrates | input | local |
| biomass | input | sub_masses.ecoli_2 |
| substrates | output | exchange |
| biomass | output | sub_masses.ecoli_2 |

#### Properties

| Property | Value |
|---|---|
| address | local.DynamicFBA |

#### Config

| Key | Value |
|---|---|
| model_file | textbook |
| kinetic_params.glucose | [1.0, 0.1] |
| kinetic_params.acetate | [0.01, 1.0] |
| substrate_update_reactions.glucose | EX_glc__D_e |
| substrate_update_reactions.acetate | EX_ac_e |
| bounds.ATPM.lower | 1.0 |
| bounds.ATPM.upper | 1.0 |
| bounds.EX_o2_e.lower | -2.0 |

## 2 Performance and comparison

This supplement evaluates the Process–Bigraph implementation of Spatio-Flux along two axes: runtime overhead and agreement with the COMETS Java backend. We ask how much computational cost is introduced by fine-grained composition, and whether the same compositional structure can expose useful parallelism for distributed execution.

### 2.1 Performance

The `Composite` runtime records two timing categories during execution. `process_update_time` measures time spent inside process `invoke()` calls. `framework_time` measures orchestration time, including projection, scheduling, reconciliation, and typed update application. These statistics are exposed through `Composite.timing_summary()`, with aggregate and per-process attribution.

We evaluated the spatio-flux testsuite, which includes single-process kinetics, dFBA communities, diffusion systems, particle simulations, and integrated reference composites. Three runtime regimes emerged. Pure dFBA workloads are process-dominated, with most runtime spent in the underlying solver. Examples include `ecoli_dfba`, `community_dfba`, and `spatial_dfba_process`, which show negligible Process–Bigraph overhead. Mixed particle–field composites divide runtime more evenly between process execution and orchestration. Highly compositional workloads with many small particle updates and deeply nested stores become framework-heavy. These cases expose the main computational cost of fine-grained compositional flexibility.

Representative examples are shown in Table 3. The timing breakdown therefore serves both as a profiling tool and as an architectural result. When runtime is dominated by biological solvers, the Process–Bigraph layer adds little measurable overhead. When runtime is framework-heavy, the results point to opportunities for batching, coarser process granularity, or distributed execution.

| **Simulation** | **Description** | **Process** | **Framework** |
|---|---|---:|---:|
| `community_dfba` | Multiple microbial dFBA models interacting through shared nutrient fields. | 99% | 1% |
| `br_particles_dfba` | Brownian particles carrying internal dFBA models coupled to environmental fields. | 52% | 48% |
| `brownian_particles` | Particle-only stochastic diffusion simulation with boundary-management processes. | 39% | 61% |
| `spatioflux_reference_demo` | Integrated metabolic, spatial, particle, and graph-rewrite composite simulation. | 34% | 66% |
| `reference_demo_x2y2` | Large-scale multi-region extension of the reference Spatio-Flux composite. | 27% | 73% |

Table 3: **Representative runtime decomposition across Spatio-Flux composites.** Percentages are normalized to process plus framework runtime. Pure dFBA simulations are dominated by solver execution, whereas highly compositional particle-based simulations spend increasing runtime in orchestration, projection, and typed update application.

#### Wall-clock scaling

Wall-clock scaling was evaluated in two settings. In both cases, the x-axis is the side length $N$ of the square lattice, so the number of simultaneous dFBA problems scales as $N^2$.

First, we compared COMETS and three local Spatio-Flux implementations on grids from $4 \times 4$ to $32 \times 32$ (Fig. 1). COMETS, run through `cometspy` [**dukowski2021comets**], serves as the reference backend. Its runtime is nearly flat at small grid sizes, indicating that fixed Java/backend costs dominate before the cost of additional dFBA solves becomes visible. At larger grid sizes, the curve begins to reflect the expected scaling with the number of dFBA solves.

The three local Spatio-Flux traces compare different ways of composing dFBA into a spatial lattice with Process–Bigraph. The per-cell `DynamicFBA` implementation uses one process node per lattice site. The `SpatialDFBA` implementation uses one larger process that loops internally over all sites. The local Ray [**moritz2018ray**] implementation dispatches per-cell work through Ray actors on the same machine. Ray is a distributed actor system that can scale across machines, while also providing modest speedups on a single machine through persistent local workers.

The three Process–Bigraph implementations start at different offsets but follow similar slopes. The gap between the per-cell `DynamicFBA` trace and the single-process `SpatialDFBA` trace measures the cost of many small process invocations relative to one larger process body. This gap is the local orchestration overhead of fine-grained composition. Importantly, the overhead appears mainly as an offset rather than a worsening scaling law. Increasing the number of process nodes does not add extra time-complexity beyond the expected growth in dFBA solves. At $32 \times 32$, COMETS remains faster than all local Spatio-Flux variants. The ordering of the Spatio-Flux traces shows how process granularity and local actor execution affect dispatch costs.

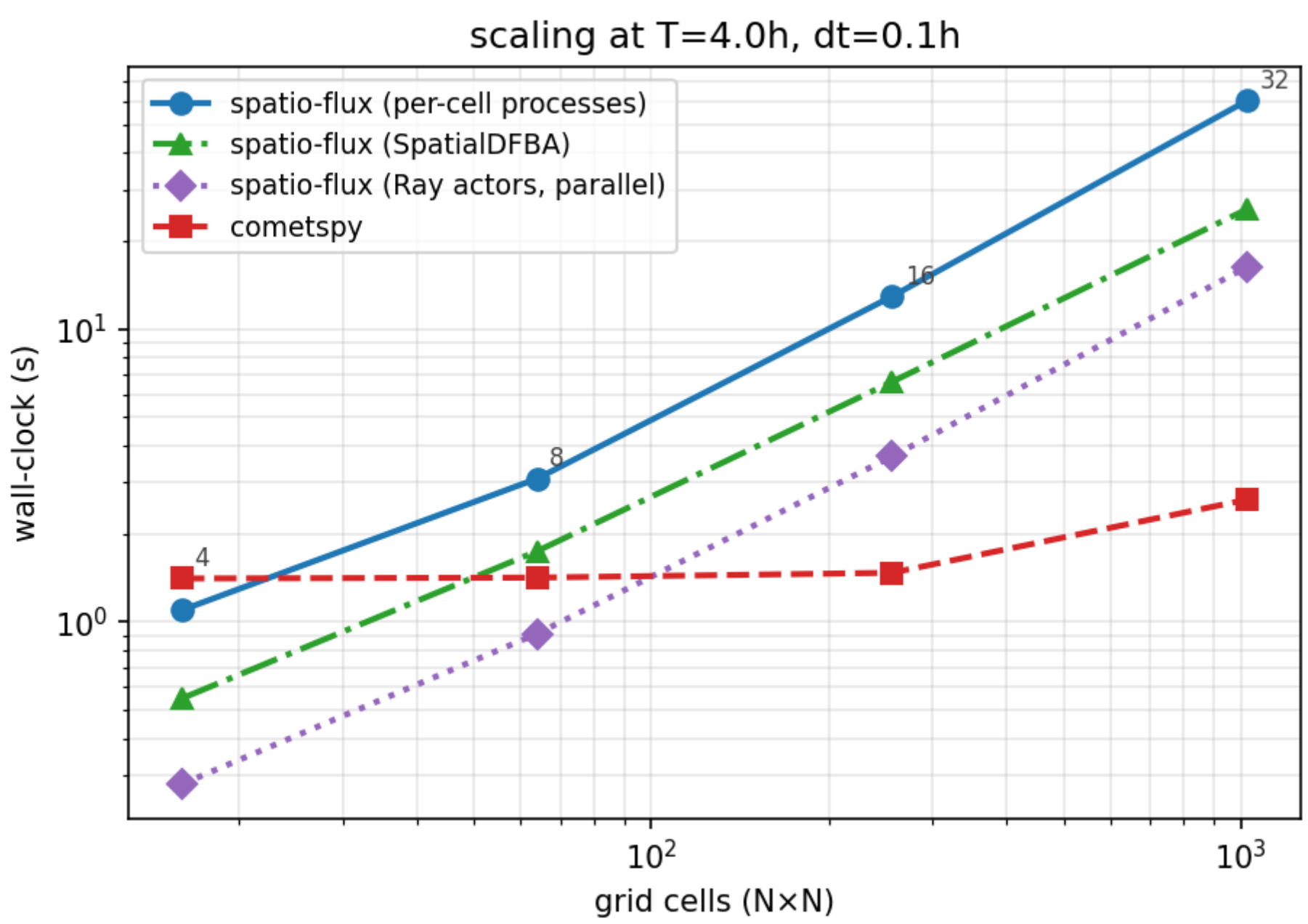


Figure 1: **Local wall-clock scaling of COMETS and Spatio-Flux execution modes.** Runtime is shown as a function of lattice side length $N$; the number of dFBA solves scales as $N^2$. COMETS is run through `cometspy` and remains nearly flat at small grid sizes because fixed Java/backend costs dominate. The three Spatio-Flux traces compare one process per lattice site, one process looping over all sites, and local Ray actors. Their comparable slopes indicate that runtime scales mainly with the number of dFBA solves, while the vertical gaps quantify orchestration overhead from finer-grained composition.

Second, we compared COMETS on an EC2 instance with distributed Spatio-Flux execution through the Ray protocol on an EC2 Ray cluster (Fig. 2). In this setting, COMETS runs on a

single machine, whereas Ray uses a head node and worker nodes. The distributed Spatio-Flux implementation partitions the lattice into shards. Each shard owns a stripe of cells and batches its assigned dFBA solves into a Ray actor call. The spatial topology does not directly constrain the dFBA solves, because inter-cell coupling is handled separately by the diffusion process operating on the full field.

This distributed mode reverses the local scaling result. Locally, many small processes add dispatch overhead. On the cluster, the same explicit decomposition exposes parallel work. Across the tested grid sizes, Ray-sharded Spatio-Flux is roughly 3–4× faster than COMETS. At $128 \times 128$, it reaches 7.2 s versus 23.6 s for COMETS. These timings exclude EC2 setup, cluster teardown, and one-time problem initialization costs in both frameworks. Those startup costs are significant, but would typically be amortized across a series of simulation runs.

Together, the local and distributed scaling results show a tradeoff. Fine-grained compositionality has a local execution cost when represented as many small Python-level processes. The same decomposition also exposes the parallel structure needed for distributed execution. Thus, the model can be developed and inspected compositionally, then scaled to distributed agents for larger spatial environments with synchronized interactions. The simulation definition, wiring, and type-directed update semantics remain unchanged; only the execution protocol changes.

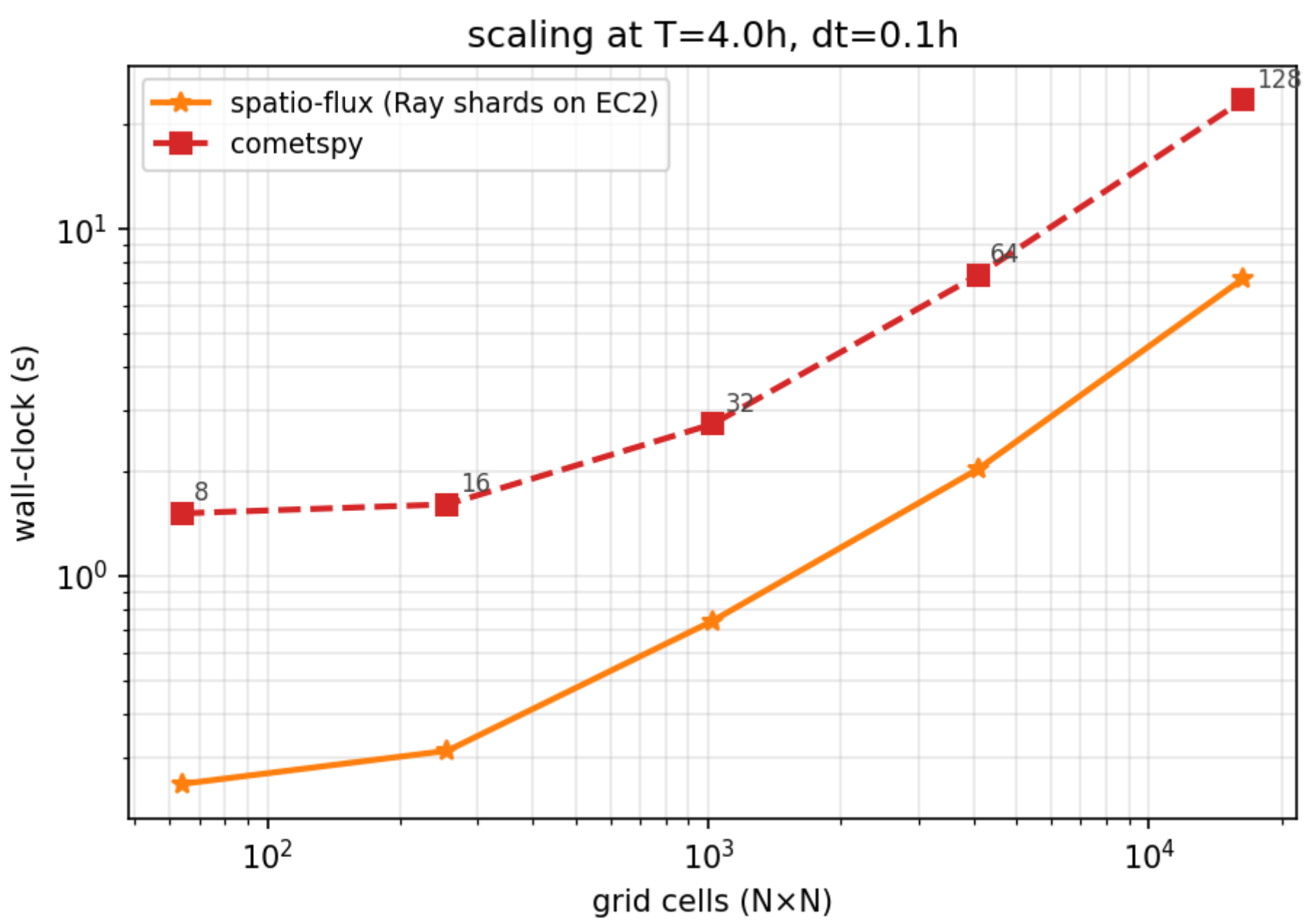


Figure 2: **Distributed wall-clock scaling of Ray-sharded Spatio-Flux against COMETS.** COMETS runs on a single EC2 instance, while Spatio-Flux uses the Ray protocol on an EC2 cluster with one head node and four worker nodes. The lattice is partitioned into shards, each batching many per-cell dFBA solves into a Ray actor call. Timings exclude EC2 setup, cluster teardown, and one-time problem initialization costs. Distributed Spatio-Flux is roughly 3–4× faster than COMETS over the tested range, reaching 7.2 s versus 23.6 s at $128 \times 128$.

**Hardware and solver context.** The local sweep was run on an AMD Ryzen 9 3900X workstation with 12 cores, 24 threads, 32 GiB RAM, Ubuntu 22.04.5, and Python 3.12.10. Local Spatio-Flux used the COBRA default GLPK solver, while COMETS used GLOP through the Java backend. The EC2 distributed sweep was run in AWS GovCloud with one m5.2xlarge head node

and four m5.4xlarge worker nodes, for 72 total vCPUs and 288 GiB RAM. In the distributed sweep, Spatio-Flux used sharded Ray actors with HiGHS-direct LP solves and warm-started simplex bases. COMETS again used GLOP on the head node. Thus, the two COMETS curves use the same Java backend and solver on different hardware, whereas the local and distributed Spatio-Flux curves use different LP backends. This solver difference contributes to small acetate-snapshot differences between the local and distributed sweeps, although biomass and glucose remain matched within approximately 1%.

## 2.2 Comparison with COMETS

We compared Spatio-Flux against the COMETS Java backend using a matched 2D dFBA-plus-diffusion benchmark implemented through `cometspy`. Both systems simulate the *E. coli* core model on an $N \times N$ lattice over four hours of simulated time. Diffusion coefficients, uptake kinetics, and environmental conditions were matched across implementations.

Each grid cell has width 0.05 cm, giving a per-cell box volume of $1.25 \times 10^{-7}$ L. The initial glucose field is a vertical gradient from 50.0 to 500.0 mM. A single biomass seed of $1.0 \times 10^{-6}$ gDW is placed at the top-center cell. Glucose and acetate diffuse with coefficient $5.0 \times 10^{-6}$ cm$^2$/s. Biomass diffusion is set to $1.0 \times 10^{-9}$ cm$^2$/s, making biomass effectively immobile over the simulated interval. Michaelis–Menten uptake kinetics are matched, with $K_m = 0.5$ mM and $V_{\max} = 1$ mmol/gDW/h for glucose. Acetate and oxygen use $V_{\max} = 2$ mmol/gDW/h.

Aligning the implementations required reconciling unit conventions and Michaelis–Menten parameter handling between Spatio-Flux and COMETS. Spatio-Flux tracks metabolite fields as mM concentrations. COMETS stores metabolites as mmol-per-box amounts and divides by box volume internally for Michaelis–Menten kinetics. The comparison code therefore converts at the `cometspy` API boundary. Concentrations are multiplied by box volume going into COMETS and divided by box volume coming back out. In addition, `cometspy`'s `defaultKm` is specified in molar rather than mM. Per-exchange `Vmax` overrides are also required for oxygen and acetate to prevent COMETS from silently clamping those exchanges to the default glucose `Vmax`. After these corrections, the implementations agreed on total biomass to within 0.1% at $N = 16$.

We evaluated four Spatio-Flux execution modes. The first uses per-cell `DynamicFBA` processes composed directly through the bigraph runtime. The second uses a monolithic `SpatialDFBA` process that loops internally over cells. The third uses local Ray actor execution. The fourth uses distributed Ray shards running across a remote EC2 cluster. The Ray protocol allows the same composite document to execute locally or remotely without modification, while preserving the same typed update semantics.

### Numerical validation

At $N = 16$, the local Spatio-Flux implementations reproduced the COMETS reference dynamics within numerical noise. Total biomass trajectories from `cometspy`, per-cell `DynamicFBA`, and `SpatialDFBA` agreed to within 0.1% (Fig. 3). Seed-cell traces likewise collapse onto a single curve after the initial transient. Glucose diffuses upward from the bottom rows, the seed cell becomes saturated, and each implementation grows at the same Michaelis–Menten-limited rate. The distributed Ray implementation remained within approximately 1% of COMETS for total biomass and within approximately 5% for local seed-cell trajectories.

Spatial snapshots showed close agreement across glucose, acetate, and biomass fields (Fig. 4). Glucose and biomass fields are visually indistinguishable across the local implementations. Acetate shows the same qualitative pattern, forming a small plume centered on the seeded biomass cell.

Minor differences in acetate peak magnitude and field shape are expected from diffusion discretization and solver choices. COMETS uses a Crank–Nicolson diffusion scheme, whereas Spatio-Flux uses an explicit FTCS scheme with CFL-bounded substepping.

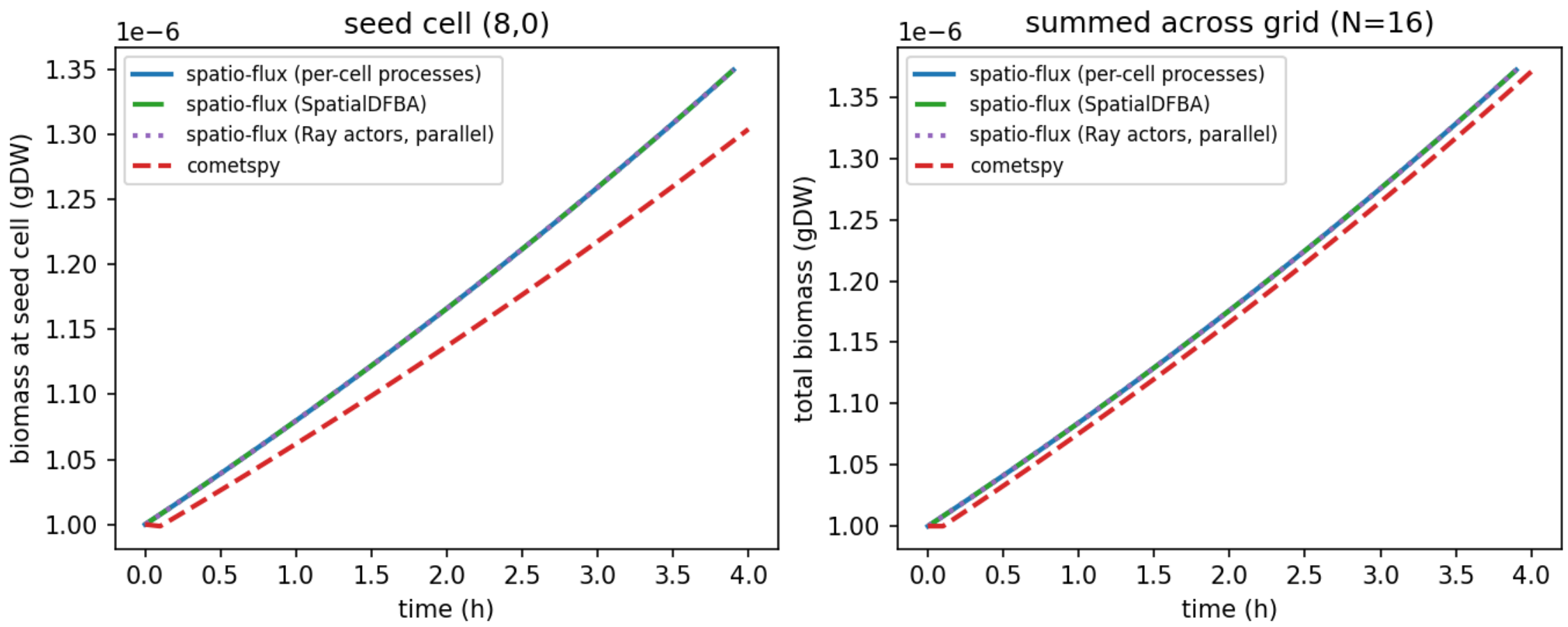


Figure 3: **Biomass trajectory comparison at** $N = 16$**.** Spatio-Flux and COMETS produce closely matching biomass dynamics for both local seed-cell biomass and total biomass across the grid.

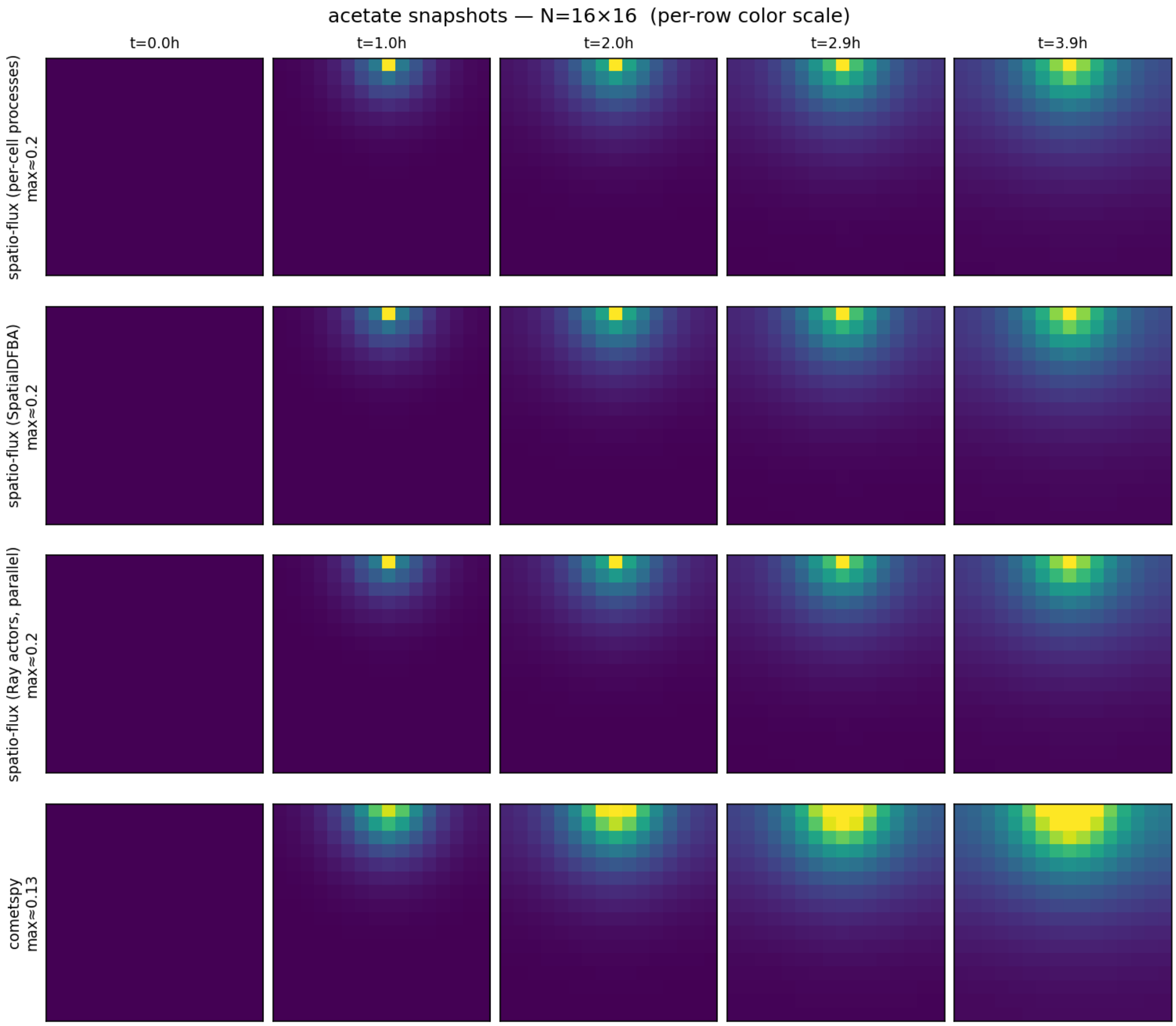


Figure 4: **Spatial acetate dynamics at $N = 16$.** Acetate secretion and diffusion patterns remain qualitatively consistent across all implementations, with minor differences arising primarily from the underlying diffusion schemes.
